# Preoperative kinesiophobia affects self‐perceived knee function and quality of life after patellar stabilising surgery

**DOI:** 10.1002/jeo2.70615

**Published:** 2026-01-08

**Authors:** Trine Hysing‐Dahl, Per Arne Skarstein Waaler, Anne Gro Heyn Faleide, Eivind Inderhaug

**Affiliations:** ^1^ Western Norway University of Applied Sciences Bergen Norway; ^2^ Haraldsplass Deaconess Hospital Bergen Norway; ^3^ Haukeland University Hospital Bergen Norway; ^4^ University of Bergen Bergen Norway

**Keywords:** Banff Patellofemoral Instability Instrument, kinesiophobia, patellar instability, rehabilitation, tampa scale of kinesiophobia

## Abstract

**Purpose:**

Kinesiophobia is an important psychosocial construct to consider in rehabilitation of patients with patellar instability, in order to optimise their rehabilitation and ability to return to sport and an active lifestyle. Therefore, it is important to investigate the percentage of patients with kinesiophobia before and 6 months after patella stabilising surgery. In addition to how it affects knee function and quality of life 6 months postoperatively.

**Methods:**

A prospective cohort of 76 patients (mean age 22.8 years, 74% female) with patellar instability was included. Patients completed patient reported outcome measures (PROMs), including the Tampa scale of kinesiophobia (TSK)‐13, International Knee Documentation Committee Subjective Knee Form (IKDC‐SKF) and Banff Patellofemoral Instability Instrument (BPII), preoperatively and 6 months postoperatively. Those with concomitant knee ligament injuries were excluded. Statistical analyses included paired sample *t*‐tests for score changes and Pearson's correlation for associations between variables.

**Results:**

Preoperative kinesiophobia was reported in 47% of patients, decreasing to 18% at 6 months postsurgery. Significant improvements were noted in all PROMs, with the BPII showing the largest increase. Patients with preoperative kinesiophobia reported worse knee function postoperatively. A strong negative correlation was found between changes in TSK‐13 and BPII scores (*r* = −0.605), indicating that reductions in kinesiophobia were associated with improvements in quality of life. In multiple regression analyse only preoperative TSK scores remained an independent significant 138 predictor of postoperative kinesiophobia, with a shared explained variance of 16%.

**Conclusion:**

This study highlights the prevalence of kinesiophobia in patients undergoing surgery for patellar instability and its effect on postoperative outcomes. While surgery restored mechanical stability, many patients continued to exhibit kinesiophobia after surgery.

**Level of Evidence:**

Level III.

**Abbreviations**
ACL: anterior cruciate ligament
BMI: body mass index
BPII: Banff Patellofemoral Instability Instrument
CI: confidence interval
IKDC‐SFK: International Knee Documentation Committee Subjective Knee Form
MPFL‐R: medial patellofemoral ligament reconstruction
PROM: patient reported outcome measures
SD: standard deviations
TSK: Tampa scale of kinesiophobia
TTO: tibial tubercle osteotomy
VAS: visual analog scale

## BACKGROUND

Patellar instability is a common knee disorder primarily affecting adolescents and young adults, with an incidence rate of 42 per 100,000 people [11] in Denmark, a population with similar demographic characteristics as Norwegian patients with patellar instability. The disorder is often accompanied by persistent pain [4, 10, 41] and functional impairment [7, 19, 41]. Alongside the physical symptoms, many patients display an increased awareness of their knee as well as adaptive behaviour in their everyday life and in sports [18] with a resultant diminished quality of life [5, 6, 20, 27, 35, 41]. Fear of new dislocations is often described prior to surgery—but also after stabilising procedures have been undertaken [28, 38]. While this fear may can be justified, arising from the perception of being prone to new dislocations or subluxations [25]—it can develop into a more general *kinesiophobia* (fear of movement).

Recent research suggests that patients with patellar instability exhibit greater kinesiophobia than healthy individuals [40] and those with other knee injuries such as an anterior cruciate ligament (ACL) tear [14]. In general, kinesiophobia can lead to a diminished sense of self‐efficacy and autonomy, as individuals may feel they are unable to participate in activities they highly value [18]. This may explain why higher degrees of kinesiophobia are associated with reduced quality of life in patients with patellar instability [14]. Meanwhile, kinesiophobia has been associated with a notable decrease in overall well‐being and prolonged rehabilitation following surgery in patients undergoing ACL reconstruction [32]. Overall, kinesiophobia is an important psychosocial construct to consider when rehabilitation patients with patellar instability, in order to optimise their rehabilitation and ability to return to an active lifestyle.

Previous studies have found that fear of new dislocations is the main reason why patients do not achieve the desired level of activity after patellar stabilising surgery [28, 38]. Furthermore, the impact of preoperative kinesiophobia on postoperative outcomes in patients with patellar instability remains unclear. No study has examined the changes in kinesiophobia after patellar stabilising surgery and how it can affect further knee function and quality of life. Therefore, this study aimed to determine the characteristics of pre‐ to postoperative changes in kinesiophobia measured by the Tampa Scale of Kinesiophobia (TSK)‐13, and its relation to self‐reported knee function and quality of life measured by the International Knee Documentation Committee Subjective Knee Form (IKDC‐SKF) and the Banff Patellofemoral Instability Instrument (BPII) 2.0, respectively. We hypothesised that: (1) both TSK‐13 scores and IKDC‐SKF/BPII scores would improve collectively from baseline to a 6‐month postoperative follow‐up; (2) the proportion of patients with evident kinesiophobia, defined as a TSK‐13 score ≥33, would decrease during the observation period; (3) patients with preoperative inferior kinesiophobia scores (≥33) would demonstrate worse knee function and quality of life than individuals without preoperative kinesiophobia at the follow‐up evaluation (4). There would be a strong correlation between IKDC score, BPII scores and kinesiophobia scores (as measured by the TSK‐13) both before and after surgery.

## MATERIALS AND METHOD

### Patient population

Patients with recurrent (>2) patella dislocations were prospectively included if they were (1) ≥13 years of age at the time of surgery, (2) fluent in Norwegian and (3) able to understand and complete the questionnaires of focus. Patients with concomitant knee ligament injuries, based on MRI findings, were excluded. All patients completed a battery of patient‐reported outcome measures (PROMs), including the TSK‐13, IKDC‐SKF and BPII, both before and 6 months after patellar stabilising surgery with an individualised approach—targeting underlying anatomic abnormalities. The study was approved by the Regional Committee for Medical and Health Research Ethics (ID: 2020/185067) and registered in ClinicalTrials.gov (NCT05119088). Written informed consent was obtained from all patients (or their legal guardians if >18 years) prior to data collection.

### PROMs

The TSK‐13 evaluates fear of movement by summarising 13 items into a total score ranging from 0 to 52, with a higher score indicating more fear of movement [31]. Although the Norwegian version of the TSK‐13 has been validated for use in patients with sciatica [13], it has also been widely used to assess kinesiophobia following knee injuries, such as ACL tears [3, 22, 36, 43]. The degree of kinesiophobia is defined after severity and described as ‘subclinical’ (13–22), ‘mild’ (23–32), ‘moderate’ (33–42) and ‘severe’ (43–52) [33]. In the current study a binary approach was chosen: ‘No kinesiophobia’ refers to subclinical/mild score, while ‘kinesiophobia’ refers to moderate/severe scores.

The IKDC‐SKF is a knee‐specific, patient‐reported tool, comprising 18 questions across three domains: symptoms, physical activity and function [21]. It yields one sum score ranging from 0 to 100 where a higher score indicates better function [21]. The IKDC‐SFK has demonstrated good psychometric properties for patients with mixed knee pathologies and injuries [2], and has been validated for patients with patellar instability [37].

The BPII evaluates quality of life through 23 items, where each item is equally weighted and answered on a visual analog scale (VAS). The final score is calculated as an average of the scores from all answered items (range 0–100), with a higher score reflecting a higher quality‐of‐life [24]. The Norwegian version is validated in patients with patellar instability, demonstrating good to excellent measurement properties in all domains [20].

### Surgical technique and postoperative rehabilitation

Prior to surgery, all patients were advised to undergo an exercise programme targeting neuromuscular deficits—with no specific focus on addressing fear of movement (if present). Type of surgery was based on findings from the preoperative counselling and radiologic examinations, including radiographs and MRI scans. All patients underwent a medial patellofemoral ligament reconstruction (MPFL‐R) by use of a gracilis autograft from the ipsilateral knee. Tibial tubercle osteotomy was considered in cases with patella alta and/or a lateralized tibial tubercle. Finally, a thin‐flap trochleoplasty procedure was considered in cases of severe trochlear dysplasia.

General advice on early neuromuscular exercises was given upon discharge from the day‐care unit, and all patients underwent postoperative rehabilitation with their local physiotherapist. Patients were not required to wear a brace and were permitted to bear weight by touching their foot to the ground, with the support of crutches, from the first postoperative day for 6 weeks. From week four after surgery, patients were permitted to gradually increase weight‐bearing until they no longer needed the crutches.

### Statistical analyses

Continuous variables are presented as mean, range, standard deviation (SD), while categorical data are presented in frequencies. Normal distribution was assessed using the Shapiro–Wilk test. A positive change, denoting an improvement in function and quality of life and reduction in fear of movement, was defined as a decrease in TSK‐13 score and an increase in BPII and IKDC‐SKF scores. The change in each score from baseline to 6 months postoperatively was analysed using a paired sample *t*‐test. An independent sample *t*‐test was conducted to examine how pre‐ and postoperative kinesiophobia affected knee function and quality of life 6 months after surgery. Percentage change in scores was calculated using the following equation:

$$\mathrm{Percentage}\;\mathrm{change}\;\left(\%\right)=\frac{\mathrm{score}\;\mathrm{at}\;6\;\mathrm{months}\;\mathrm{postoperatively}-\mathrm{preoperative}\;\mathrm{score}}{\mathrm{preoperative}\;\mathrm{score}}*100.$$

Correlations were investigated using Pearson's *r*; 0.10–0.29 was considered *small*, 0.30–0.49 *medium* and 0.50–1.0 *large* [8, p. 79–81]. The a priori significance level was set to ≤0.05, with Bonferroni‐corrections for multiple comparisons. To examine which factors that predict performance, multiple regression was performed. With kinesiophobia 6 months postoperative as the dependent variable, age, duration of symptoms, type of surgery and preoperative TSK was entered as independent variables, and only variables with a *p*‐value ≤ 0.10 were included in the final model. Multicollinearity was assessed by inspecting the tolerance values in linear regression analysis, and values < 0.1 were interpreted to indicate correlations that are too high between variables. The statistical analyses were performed using the IBM SPSS Statistics for Windows, version 26.0 (IBM Corp). A post‐hoc group size calculation was performed based on former studies with a standard deviation of BPII of 16.6 and MICD of 6.2 [14, 24]. With a statistical significance of 0.05, a *β*‐value of 0.1 (power of 0.9), a group size of 76 was found to be sufficient.

## RESULTS

A total of 76 patients, 74% females with a mean age of 22.8 years, met the inclusion criteria for this study. Demographic data can be found in Table 1.

**Table 1:** Patient characteristics (*n* = 76).^a^

Age at surgery, years	22.8 ± 7.5
Female gender	56 (73.7)
Years since first dislocation (range)	7.5 ± 6.7 (0‐20)
Bilateral patellar instability	46 (59.7)
BMI	24.9 ± 5.9
Surgical procedure	
MPFL‐R	11 (14.5)
MPFL‐R + TTO	36 (47.4)
MPFL‐R + trochleoplasty	17 (22.4)
MPFL‐R + TTO and trochleoplasty	12 (15.8)

Abbreviations: BMI, body mass index; MPFL‐R, medial patellofemoral ligament reconstruction; TTO, tibial tubercle osteotomy.

^a^ Data are reported as *n* (%) or mean ± SD.

Of these, 36 (47%) reported preoperative kinesiophobia (defined as TSK‐13 scores ≥33 points) whereas 6 months after surgery only 14 (18%) patients exceeded the threshold for kinesiophobia. There was a significant improvement in all PROMs scores from pre‐ to postoperative assessments, where mean BPII score displayed the largest numeric improvement (Table 2).

**Table 2:** Changes in PROM scores from pre‐ to 6 months postoperatively (*n* = 76).

Test	Preoperative (SD)	6 months postoperative (SD)	*p*‐Value^a^	Mean change (95% CI)	Percentage change (%)
TSK	32.6 ± 6.9	25.9 ± 6.2	**<0.001**	−6.75 (−8.5 to −5.0)	−18.9 ± 2.3
IKDC‐SKF	57.3 ± 15.4	68.4 ± 15.1	**<0.001**	11.1 (7.5–14.6)	15.2 ± 3.3
BPII	42.4 ± 17.5	64.8 ± 19.7	**<0.001**	22.4 (18.3–26.4)	33.3 ± 2.8

*Note*: Data are reported as mean ± SD. Bolded *p*‐value indicates a statistically significant difference between groups (*p* < 0.05).

Abbreviations: BPII, The Banff Patellofemoral Instability Instrument 2.0; IKDC‐SKF International Knee Documentation Committee Subjective Knee Form; TSK, Tampa scale of kinesiophobia.

^a^ Bonferroni adjustment for multiple comparisons.

The extent of surgery affected the preoperative TSK sores and the percent changes in TSK scores from pre‐ to postoperative (Table 3).

**Table 3:** Difference in TSK scores in patients with MPFL‐R and MPFL‐R with concomitant procedures (*n* = 76).

	MPFL‐R (*N* = 11)	MPFL‐R with concomitant procedures (*N* = 65)	*p‐*Value^a^	Effect size (95% CI)
TSK preoperative	27.6 ± 6.2	33.7 ± 6.4	**0.004**	−0.96 (−1.62 to 0.30)
TSK postoperative	26.9 ± 8.3	25.8 ± 5.8	0.297	0.18 (−0.47 to 0.82)
% changes in TSK	4.0 ± 15.9	21.4 ± 19.1	**0.006**	0.93 (0. 27 to 1.58)

*Note*: Data are reported as mean ± SD, 95% CI. Bolded *p*‐value indicates a statistically significant difference between groups (*p* < 0.05).

Abbreviation: TSK, Tampa scale of kinesiophobia.

^a^ Bonferroni adjustment for multiple comparisons.

Patients with preoperative kinesiophobia had worse self‐reported knee function 6 months postoperatively compared to individuals without kinesiophobia preoperatively, no such effect were found on quality of life (Table 4).

**Table 4:** Difference in postoperative scores in patients with and without preoperative kinesiophobia (*n* = 75).

Test	No kinesiophobia (*N* = 40)	Kinesiophobia (*N* = 36)	*p*‐Value^a^	Effect size (95% CI)
IKDC‐SFK	71.8 ± 15.7	64.8 ± 13.3	**0.004**	0.69 (0.23 to 1.16)
BPII	67.2 ± 21.1	62.2 ± 17.9	0.264	0.26 (−0.20 to 0.71)

*Note*: Data are reported as mean ± SD. Bolded *p*‐value indicates a statistically significant difference between groups (*p* < 0.05).

Abbreviations: BPII, The Banff Patellofemoral Instability Instrument 2.0; IKDC‐SKF, International Knee Documentation Committee Subjective Knee Form.

^a^ Bonferroni adjustment for multiple comparisons.

There was a large association between percent change in TSK‐13 and BPII (*r* = −0.605) whereas no significant association was found between changes in IKDC‐SKF and TSK‐13 (Table 5).

**Table 5:** Correlation between % changes in kinesiophobia and changes in BPII and IKDC‐SKF scores (*n* = 76).

Measurement	% change from pre‐ to postoperative	Pearsons *r*	*p*‐value
TSK	18.6		
IKDC‐SKF	16.5	−0.180	0.120
BPII	32.7	−0.605	**<0.001**

*Note*: Bolded *p*‐value indicates a statistically significant correlation between tests (*p* < 0.05).

Abbreviations: BPII, The Banff Patellofemoral Instability Instrument 2.0; IKDC‐SKF International Knee Documentation Committee Subjective Knee Form.

Prescence of *kinesiophobia* before surgery demonstrated large associations with both knee function and quality of life before surgery (Table 6). Postoperative kinesiophobia was largely associated with quality of life, and only moderate associated to knee function 6 months after surgery (Table 6).

**Table 6:** Correlation between TSK‐13 and IKDC‐SKF and BPII (*n* = 76).

Measurement	Correlation between preoperative scores		Correlation between postoperative scores	
	Pearsons *r*	*p*‐Value	Pearsons *r*	*p*‐Value
IKDC‐SKF	−0.553	**<0.001**	−0.328	**0.004**
BPII	−0.701	**<0.001**	−0.538	**<0.001**

*Note*: Bolded *p*‐value indicates a statistically significant correlation between tests (*p* < 0.05).

Abbreviations: BPII: The Banff Patellofemoral Instability Instrument 2.0; IKDC‐SKF International Knee Documentation Committee Subjective Knee Form; TSK‐13: Tampa scale for kinesiophobia.

In the multiple regression model, only preoperative TSK scores remained an independent significant predictor of postoperative kinesiophobia, with a shared explained variance of 16% (Table 7). Tolerance value was 0.90, indicating no problems with multicollinearity.

**Table 7:** Predictors of postoperative kinesiophobia.

Dependent variable	Independent variables	Beta	*p*‐Value^a^	*R* ^2^
Postoperative TSK	Age	0.042	0.795	
	Duration of symptoms	−0.267	0.104	
	Type of surgery	−0.040	0.720	
	Preoperative TSK	0.386	0.001	0.160

*Note*: Final multiple regression models with gender, age, duration of symptoms, type of surgery and preoperative TSK score as covariates (*N* = 76).

Abbreviation: Tampa scale of kinesiophobia.

^a^ Independent variables predicting postoperative kinesiophobia with *p* ≤ 0.10 were retain in the final model.

## DISCUSSION

The main finding of the current study was that presence of preoperative kinesiophobia has a negative effect on PROMs 6 months after patellar stabilising surgery, where patients displaying kinesiophobia before surgery had smaller magnitude of improvement compared to the patients without preoperative kinesiophobia. Overall, there was a significant improvement in both self‐reported knee function and quality of life at 6 months after surgery—with a clear reduction in the number of patients demonstrating kinesiophobia compared to the preoperative setting.

The fact that almost half of all the patients in the current population displayed significant kinesiophobia (defined as 33 and above on TSK) before surgery reflects what has previously been reported in both qualitative [18] and quantitative [14, 40] studies in this group of patients. One could argue that fear of movement is a sound reaction to the unpredictability of living with patellar instability (i.e. never knowing when your patella would dislocate). Furthermore, after restoration of mechanical knee stability—through surgery and following rehabilitation ‐ most patients seem to regain trust in their knee and thereby reduces degree of kinesiophobia as demonstrated by the major reduction in number of patients with kinesiophobia 6 months after surgery (47% preoperatively vs. 18% postoperatively). Nevertheless, one fifth of patients is still a considerable number of patients and implies that rehabilitation needs to consider this construct. There is therefore a need for including targeted cognitive interventions and to address the fear of new dislocations throughout the entire trajectory of rehabilitation, both preoperatively as well as the later phases. However, how this should be done is currently undecided [17]. Lessons could potentially be drawn from interventions suggested for similar knee injuries, such as ACL tears. These include patient education, gradual exposure, regular testing and cognitive behavioural therapy when needed [23].

It is also worth noting how kinesiophobia is demonstrated to influence recovery and prolong rehabilitation in patients with other knee problems [1, 3, 12, 22]. Similar, in the current cohort, patients with preoperative kinesiophobia demonstrated worse knee function and quality of life after surgery compared to individuals without kinesiophobia. However, all patients had some improvement in knee function and quality of life after patellar surgery—is in line with most other studies reporting on effect of treatment [7, 34, 35, 42, 44, 45, 47]. These findings support the need to deliver additional support to patients with preoperative kinesiophobia as described above. Physiological responses (e.g., fear of movement) will always occur in physical trauma or injury and most patients will experience some negative emotions, and lack of self‐confidence, because of reduced knee function [22]. These changes will most likely be lowered throughout the course of rehabilitation as patients improves function and regains trust in their knee [18]. Therefore, it is not surprising that the current changes in kinesiophobia seem to be highly associated with changes in quality of life (*r* = −0.605). The negative relationship is due to the inverse nature of the scores, therefore, when fear of movement reduces, quality of life increases. The strong correlations between kinesiophobia and quality of life both before and after surgery for patellar instability (*r* = −0.701 and −0.538) are in accordance with research on patients undergoing ACL reconstruction [22, 29]. A more surprising finding was that changes in knee function did not correlate with changes in kinesiophobia. The observed outcomes may be attributable to the nature of IKDC‐SKF as a joint‐specific, rather than diagnosis‐specific, PROM, thereby not capturing all aspects of knee function in patients with PI [15]. This is supported by indications of poor content validity for the population with PI [37]. Furthermore, it might be taken as support for the notion that knee function and kinesiophobia are only partly overlapping constructs ‐ as demonstrated by the large association before surgery (*r* = −0.553) and only moderate association after surgery (*r* = −0.328). In addition, contrasting results regarding the correlation between kinesiophobia and knee function after ACL injury have been demonstrated in the literature [29]. These findings call for more research to elaborate the current results.

The reported return to sport (after surgery) rates for patellar instability patients in current literature span from 50% to 100% [26, 28, 30, 38, 39]. Across all studies, the primary factor for not returning to sport, were psychological reasons—defined as mental barriers, lack of psychological readiness and/or reduced confidence in the knee [16, 28, 30, 38]. Knowing that kinesiophobia reduces the probability for returning to sport after ACL reconstruction [46] it is reasonable to assume that the same factor also plays a role in the current patient group of focus. There is, however, only a scarce base of knowledge on this issue. Therefore, it would be interesting to evaluate the timing of surgery—if early surgical interventions, for example, after the first dislocation episode would have effect on the degree of kinesiophobia. It is also interesting to examine when targeted psychological intervention should be introduced during the rehabilitation course.

There are some inherent limitations in this study; although the TSK has formerly been used to measure fear of re‐injuries in patients undergoing MPFL reconstruction, it was originally developed to assess fear of movement in patients with low back and might therefore lack some validity for the current group [40]. Second, the existing Norwegian version of the IKDC‐SKF, which was used in the present study, has not undergone the recommended assessment of its measurement properties [9]. Despite some limitations in information available on the measurement properties of the TSK and IKDC‐SKF, they are extensively used and widely accepted within the research and clinical communities. Thirdly, the study included patients undergoing a spectrum of procedures, ranging from isolated MPFL‐R to trochleoplasty and tuberosity tibia osteotomy. Length of rehabilitation may differ between these procedures, and it should be recognised that this might have affected the PROMs at the 6‐month follow‐up. However, a previous study found no difference in patient performance 6 months after surgery, regardless of the extent of the procedure [19]. Finally, specific details of the rehabilitation program were not collected. Variations in adherence may interfere with outcomes in this study. Nevertheless, results from the current study should therefore be interpreted with these factors in mind.

## CONCLUSION

A significant proportion of patients in the current cohort, undergoing surgery for patellar instability, exhibit symptoms of kinesiophobia. This appears to have a negative influence on postoperative outcomes. However, both self‐reported knee function and quality of life improved until 6 months after surgery. The degree of kinesiophobia decreased postoperatively, and the extent of this decrease was highly associated with the changes in quality of life. This study describes how restoring mechanical stability of the patella allows for a reduction in the fear of movement, yet predisposed individuals still display kinesiophobia 6 months after surgery. These results highlight the need to include early and targeted psychological interventions throughout the rehabilitation process.

## CLINICAL RECOMMENDATIONS

Due to the high number of patients displaying kinesiophobia, rehabilitation should include psychological interventions both before and after patella stabilising surgery.

## AUTHOR CONTRIBUTIONS

**Trine Hysing‐Dahl**: Conception and design of study; acquisition of data; analysis and interpretation of data and drafting of manuscript. **Per Arne Skarstein Waaler**: Conception design of study and critically revising manuscript. **Anne Gro Heyn Faleide**: Conception design of study and critically revising manuscript. **Eivind Inderhaug**: Conception and design of study and critically revising manuscript.

## CONFLICT OF INTEREST STATEMENT

The authors declare no conflicts of interest.

## ETHICS STATEMENT

The study was approved by the NSD (Norwegian Centre for Research Data) Data Protection Official for Research, project number 731409 and the Regional Committee for Medical and Health Research Ethics (ID: 2020/185067).

## Data Availability

Data are available on reasonable request.
